# Intravital imaging reveals systemic ezrin inhibition impedes cancer cell migration and lymph node metastasis in breast cancer

**DOI:** 10.1186/s13058-018-1079-7

**Published:** 2019-01-24

**Authors:** Abdi Ghaffari, Victoria Hoskin, Gulisa Turashvili, Sonal Varma, Jeff Mewburn, Graeme Mullins, Peter A. Greer, Friedemann Kiefer, Andrew G. Day, Yolanda Madarnas, Sandip SenGupta, Bruce E. Elliott

**Affiliations:** 1 Department of Pathology and Molecular Medicine, Queen’s University, Kingston, Canada; 2 Max Planck Institute for Molecular Biomedicine, Münster, Germany; 3 Department of Oncology, Queen’s University, Kingston, Canada; 4 Cancer Research Institute, Division of Cancer Biology and Genetics, Queen’s University, 18 Stuart Street, Kingston, ON K7L 3N6 Canada; 5 Kingston General Hospital Research Institute, Kingston, Canada

**Keywords:** Ezrin, Quantitative intravital imaging, Cell migration, Lymph node metastasis, Biomarker, Metastatic disease

## Abstract

**Background:**

Limited understanding of the cancer biology of metastatic sites is a major factor contributing to poor outcomes in cancer patients. The regional lymph nodes are the most common site of metastasis in most solid cancers and their involvement is a strong predictor of relapse in breast cancer (BC). We have previously shown that ezrin, a cytoskeletal–membrane linker protein, is associated with lymphovascular invasion and promotes metastatic progression in BC. However, the efficacy of pharmacological inhibition of ezrin in blocking cancer cell migration and metastasis remains unexplored in BC.

**Methods:**

We quantified ezrin expression in a BC tissue microarray (*n* = 347) to assess its correlation with risk of relapse. Next, we developed a quantitative intravital microscopy (qIVM) approach, using a syngeneic lymphatic reporter mouse tumor model, to investigate the effect of systemic ezrin inhibition on cancer cell migration and metastasis.

**Results:**

We show that ezrin is expressed at significantly higher levels in lymph node metastases compared to matched primary tumors, and that a high tumor ezrin level is associated with increased risk of relapse in BC patients with regional disease. Using qIVM, we observe a subset of cancer cells that retain their invasive and migratory phenotype at the tumor-draining lymph node. We further show that systemic inhibition of ezrin, using a small molecule compound (NSC668394), impedes the migration of cancer cells in vivo. Furthermore, systemic ezrin inhibition leads to reductions in metastatic burden at the distal axillary lymph node and lungs.

**Conclusions:**

Our findings demonstrate that the tumor ezrin level act as an independent biomarker in predicting relapse and provide a rationale for therapeutic targeting of ezrin to reduce the metastatic capacity of cancer cells in high-risk BC patients with elevated ezrin expression.

**Electronic supplementary material:**

The online version of this article (10.1186/s13058-018-1079-7) contains supplementary material, which is available to authorized users.

## Background

Cancer is not only a disease of uncontrolled growth, but in its most aggressive form it is also a disease of uncontrolled cell migration. Ezrin, a member of the ezrin–radixin–moesin (ERM) family of actin cytoskeleton–plasma membrane linker proteins, is involved in multiple aspects of cancer cell migration and its overexpression has been associated with poor prognosis in a variety of solid tumors [1]. Ezrin plays a critical role in epithelial morphology, adhesion, and migration - all key events that contribute to the invasive phenotype of cancer cells during tumor progression [2]. It is therefore not surprising that genetic ablation of ezrin or mutation of its key binding sites has been shown to impair metastatic progression in experimental models of a variety of solid cancers [3].

Lymph nodes (LNs) are the most common sites of solid tumor metastases, and spread of cancer cells to regional LNs is one of the strongest predictors of risk of relapse in BC patients [4]. In BC, there is extensive evidence for preferential dissemination of cancer cells via the lymphatic rather than blood vascular route [5]. Furthermore, the addition of regional nodal irradiation to conventional treatment, after mastectomy or breast-conserving surgery in node-positive patients, reduces the rate of locoregional and systemic recurrence [6, 7]. Despite this clinical importance, and partly due to a lack of appropriate single-cell resolution imaging models of LN metastasis, we have a very limited understanding of the fate of cancer cells within LNs and of the efficacy of therapeutic targeting of prometastatic molecules to prevent further metastatic spread of cancer cells beyond regional LNs. We have previously shown that tumor ezrin levels correlate with lymphovascular invasion in a locally accrued BC cohort and that ezrin acts cooperatively with Src in regulating tumor lymphangiogenesis [8]. Clinically, ezrin overexpression has also been reported to correlate with the presence of lymph node metastasis in breast cancer [9], pancreatic cancer [10], and nonsmall-cell lung cancer [11]. Together, these findings prompted us to examine whether pharmacological inhibition of ezrin could have therapeutic benefits by suppressing the spread of highly metastatic cancer cells from lymph node micrometastases.

Ezrin’s interactions are dependent on conformational activation of the molecule. In its inactive state, ezrin binding sites are masked by intramolecular interaction between its N-terminal 4.1/ezrin/radixin/moesin (FERM) domain and the C-terminus. Upon phosphorylation of a conserved C-terminal threonine residue (T567) by protein kinase C or Rho kinase and subsequent binding with membrane-associated phosphatidylinositol 4,5-biphosphate (PIP2), the intramolecular masking of its binding sites is removed and interaction with binding partners can take place [2]. Bulut and colleagues have recently discovered several small molecule inhibitors with high binding affinity to ezrin and their ability to block T567 phosphorylation [12–14]. These authors demonstrated a reduction in the invasive phenotype of cancer cells and inhibition of lung metastases in mice treated with ezrin inhibitors in osteosarcoma experimental models [12].

In this study, we have shown that elevated tumor ezrin protein expression is associated with increased risk of relapse in node-positive and high-risk node-negative BC patients. We developed a novel qIVM approach using tumor-bearing lymphatic reporter mice to track metastatic cancer cell migration in vivo in real time. We observed a significant reduction in migration and invasion capacity of cancer cells in tumor-draining inguinal lymph node metastases following pharmacological inhibition of ezrin, with a concomitant decrease in metastatic burden in the draining axillary node.

## Methods

### Breast cancer patient cohort and tissue microarray

A tissue microarray (TMA) (*n* = 347) was constructed from unselected archival (formalin-fixed paraffin-embedded) breast tumor specimens from consenting patients treated at the Cancer Centre of Southeastern Ontario at Kingston General Hospital (SEOBC cohort) between 1996 and 2007 (*n* = 450). A summary of clinicopathological data is presented in Additional file 1: Figure S1A. Written informed consent was obtained from all patients and the studies were conducted in accordance with the Queen’s University Research Ethics Board consent guidelines. Patients with previous history of cancer, bilateral disease, or neoadjuvant chemotherapy were excluded. Archival reduction mammoplasties from consenting patients were included as nonmalignant controls. Each sample was represented by triplicate cores (0.6 mm) from the tumor and adjacent benign tissue, carefully annotated by two pathologists (SV, SSG). Immunohistochemistry (IHC) staining of ER, PR, HER2, Ki-67, EGFR, and CK5/6 was performed at the Centre for Translational and Applied Genomics (BC Cancer Agency, Vancouver, BC, Canada) and scored visually by a pathologist (GT). High-risk node-negative BC is defined as patients with tumor size larger than 1.0 cm and one or more of the following parameters: tumor grade ≥ 3, negative ER status, and positive lymphovascular invasion (LVI). Ezrin gene expression data were obtained from 844 breast cancer patients enrolled in The Cancer Genome Atlas (TCGA) study as described previously and based upon data generated by the TCGA Research Network (http//cancergenome.nih.gov) [15]. Normalized RNA-Seq data (version 2, level 3) were used to analyze ezrin gene expression in benign and breast tumor tissues.

### Immunohistochemistry

IHC was performed as previously described [15]. Biomarker (ezrin) staining and analysis on our BC cohort was performed according to REMARK guidelines [16]. In brief, freshly cut 5 μm thick TMA, whole tissue sections, or mouse tumour tissue were stained with antibodies against ezrin (Sigma-Aldrich, Oakville, ON; cat# E8897, clone 3C12), and AE1/AE3 cytokeratin (Santa Cruz Biotechnologist, Dallas, Texas; cat# sc-81,714) antibodies using the automated Ventana Discovery XT staining system (Ventana Medical Systems, Tucson AZ) and EDTA buffer for antigen retrieval process (pH 8.0, 100 °C). Ezrin-expressing and ezrin-deficient cell pellets were included in the TMA as positive and negative controls. Technical reproducibility of ezrin stain was assessed by comparing replicate staining of serial sections from whole tumour tissue as well as a test BC TMA. Tumour tissues harvested from mice were processed for IHC staining by Ventana system using lyve-1 (Millipore Sigma, Etobicoke, ON, cat# AB2988) and CD31 (Santa Cruz, cat# sc-1506) antibodies as previously described [8]. Number of lymphatic (lyve-1^high^, CD31^low^) and blood (lyve-1^low^, CD31^high^) vessels were quantified (manual count by students blinded to the study) in at least 5 random fields of view (200X magnification) in peritumoral regions.

### TMA automated scoring

TMA slides were scanned by ScanScope (Aperio Technologies, Vista, CA, USA) after IHC to obtain the digital images. Automated scoring of ezrin IHC stains was performed as described previously [15]. In brief, the HALO (Indica Labs Inc., Corrales, NM, USA) algorithm was optimized, under pathologist supervision, using a cytoplasmic/membrane script, which gates hematoxylin-stained tumor nuclei based on size, shape, compactness, and roundness. This, in combination with manual annotations and a classifier that recognized the stromal pattern, allowed for scoring ezrin stains at a single-cell resolution in only tumor regions of TMA cores. The HALO output summarizes the percentage of tumor cells in each core that stained negative, weak, moderate, and strong for ezrin expression. A histopathology score (H-score) was then calculated for each core by multiplying percent positive cell at each staining intensity (a value from 0-300) and expressed as the average of 3 cores per tumor/patient. Cores with less of 50 tumor cells were excluded from the analysis.

### Cell lines and plasmids

EO771 medullary breast adenocarcinoma cells were purchased in 2014 (cat# 940001; CH3 BioSystems, Amherst, NY, USA), and were originally isolated by Dr FM Sirotnak (Memorial Sloan-Kettering Cancer Centre, New York, NY, USA) from a spontaneous cancer in C57BL/6 mice [17]. Orthotopic and subcutaneous syngeneic tumors derived from the EO771 cell line were described previously [18, 19]. EO771 cells were cultured in DMEM with 10% FBS (Sigma-Aldrich) and 1% glutamine supplement. EO771 cells were transduced with GFP-expressing ecotropic lentivirus containing pWPXLD plasmid using Polyjet (Froggabio, North York, ON Canada) according to the manufacturer’s instructions. A highly metastatic variant of the GFP-EO771 cell line was selected as described previously with some modifications [20]. GFP-EO771 cells (5 × 10^5^ cells) suspended in Matrigel/PBS (50:50) were injected into the mammary fat pad (MFP) of female C57BL/6 mice using a Hamilton syringe. Four weeks later, mice were dissected and examined for established lung metastases using a dissecting microscope. A single lung nodule was dissected, minced, and engrafted into the MFP of a female C57BL/6 mouse using a 16-gauge needle. This process was repeated three times, with the final metastatic isolates cultured in growth media for the generation of a stable lung metastatic variant (LMV) of GFP-EO771 cell line (EO771^LMV^). All animal procedures were carried out according to the guidelines of the Canadian Council on Animal Care with the approval of the Queen’s University Animal Care Committee. MDA-MB-231 human BC cells were obtained from Dr P Siegel in 2007 (McGill University, Montreal, QC, USA) and transfected with human Ezrin shRNA or empty vector control pLKO.1 lentiviral vector as described previously [20]. All cultured cell lines were used before passage 10 from the original frozen stocks and routinely tested for mycoplasma (Lookout Mycoplasma PCR Detection Kit, Sigma Aldrich) and found to be free of contamination.

### Immunoblotting

Whole-cell lysates were prepared as described previously [20]. Total protein concentrations were determined using the DC Protein Assay (Bio-Rad, Mississauga, ON, Canada). Lysate proteins (10–20 μg) were separated by SDS-PAGE, transferred to 0.45-μm PVDF membranes (EMD Millipore, Etobicoke, ON, Canada), blocked in 5% nonfat dry milk in 1× Tris-buffered saline/0.1% Tween-20, and then probed with rabbit anti-ezrin (cat# 3145; Cell Signaling), anti-phospho-threonine Ezrin/Radixin/Moesin (pTERM, cat# 3149; Cell Signaling), and anti-γ-tubulin (cat# T5326; Sigma-Aldrich).

### Real-time in-vitro cell migration assay

GFP-EO771^LMV^ cells were seeded (2 × 10^4^ cells) sparsely onto collagen-coated four-well μ-slides (Ibidi, Madison, WI, USA) in DMEM plus 10% FBS and 20 mM HEPES, and incubated at 37 °C for 2 h. Cells were then imaged every 10 min for 15 h using a 10× objective on an inverted Quorum WaveFX-X1 Spinning Disk confocal microscope equipped with a closed chamber at 37 °C and 5% CO_2_. Individual cells were tracked using MetaMorph software by two observers blinded to the study. Cell tracking and directional migration of cell migration were analyzed using the open source DiPer program [21] and Excel. A minimum of 30 cells were tracked per experiment.

### Intravital imaging of lymph node metastasis

All animal procedures were carried out according to the guidelines of the Canadian Council on Animal Care with the approval of the Queen’s University Animal Care Committee. Female prox1-mOrange2-pA-BAC lymphatic reporter mice (8–10 weeks old), developed by Dr F Kiefer and colleagues [22], were injected subcutaneously into the left flank with GFP-EO771^LMV^ cells (1 × 10^5^ cells). When tumors reached 400 mm^3^ (~ 20 days), mice were anesthetized with ketamine (200 mg/kg) and xylazine (10 mg/kg) and a jugular vein catheter was inserted for intravenous delivery of anesthetic during the procedure. Skin flap surgery was performed to expose the inguinal LN by careful removal of adipose and connective tissue. The skin flap was then stabilized on a heated motorized microscope stage using holding clamps, a 2 cm × 2 cm piece of foam, and surgical tape to avoid tissue drift and compression during imaging. Intravital imaging was performed on a Confocal Quorum WaveFX-X1 spinning-disk microscope (Quorum Technologies, Guelph, Canada) equipped with a Hamamatsu EMCCD camera (Hamamatsu, Japan), based on an imaging system previously described by Dr Paul Kubes’ group [23], which allowed for simultaneous visualization of GFP-expressing cancer cells (Ex 491 nm), prox1-mOrange2-expressing lymphatic endothelial cells (LECs) (Ex 561 nm), and eFluor660-CD3-labeled T cells (Ex 642 nm; eBioscience, San Diego, CA, USA). To visualize blood vasculature, Alexa647-CD31 antibody (BioLegend, San Diego, CA, USA) was injected intravenously (200 μl) using the catheter prior to imaging. To track cell motility in TDLN or trafficking within lymphatic vessels, 4D images were acquired with a 20× objective every 30–60 s for up to 2.5 h with 2-μm z-stacks spanning 4–20 μm from the focal plane and a maximum depth of 100 μm into the inguinal LN cortex. Images were analyzed in Metamorph software (Molecular Devices, Sunnyvale, CA, USA) with minimal processing to reduce noise and adjust brightness/contrast. GFP-expressing cancer cells were manually tracked in Metamorph Software by two students blinded to the study. Cell track data were graphed in Microsoft Excel using the DiPer macro method described previously [21]. The number of fields per LN, number of mice, and number of experiments are indicated in the appropriate figure legends.

### Assessment of lung metastasis in EO771 orthotopic tumor model

GFP-EO771^LMV^ cells (5 × 10^5^ cells) suspended in Matrigel/PBS (50:50) were injected into the (fourth) mammary fat pad of female C57BL/6 mice using a Hamilton syringe. When tumors reach a palpable size (~ 100 mm^3^), mice received daily treatment of ezrin inhibitor NSC668394 (0.5 mg/kg, i.p.) or vehicle (0.01% DMSO/PBS) until tumors reached ~ 1 cm in diameter (~ day 20). At this point, primary tumors (~ 1 cm diameter) were surgically removed and mice were allowed to recover. Lung metastases were allowed to expand for 1 week more prior to harvesting and assessment of total lung tumor nodules by fluorescence imaging.

### Statistical analysis

All statistical analyses were performed using SPSS or GraphPad Prism software, unless otherwise indicated. Data are presented as mean ± SD and *p* < 0.05 was considered significant. Specific statistical tests are described in the figure legends. In brief, the *p* values were calculated by Student’s *t* test or Mann–Whitney *U* test between two means and by Kruskal–Wallis test followed by Dunnett’s multiple comparison tests for three or more means. The log-rank test was used to assess statistical significance between Kaplan–Meier disease-free survival curves. Statistical analyses of clinical outcome were performed under supervision of the team’s biostatistician (AGD).

## Results

### High tumor ezrin levels correlate with increased risk of relapse in invasive BC

**Fig. 1:**
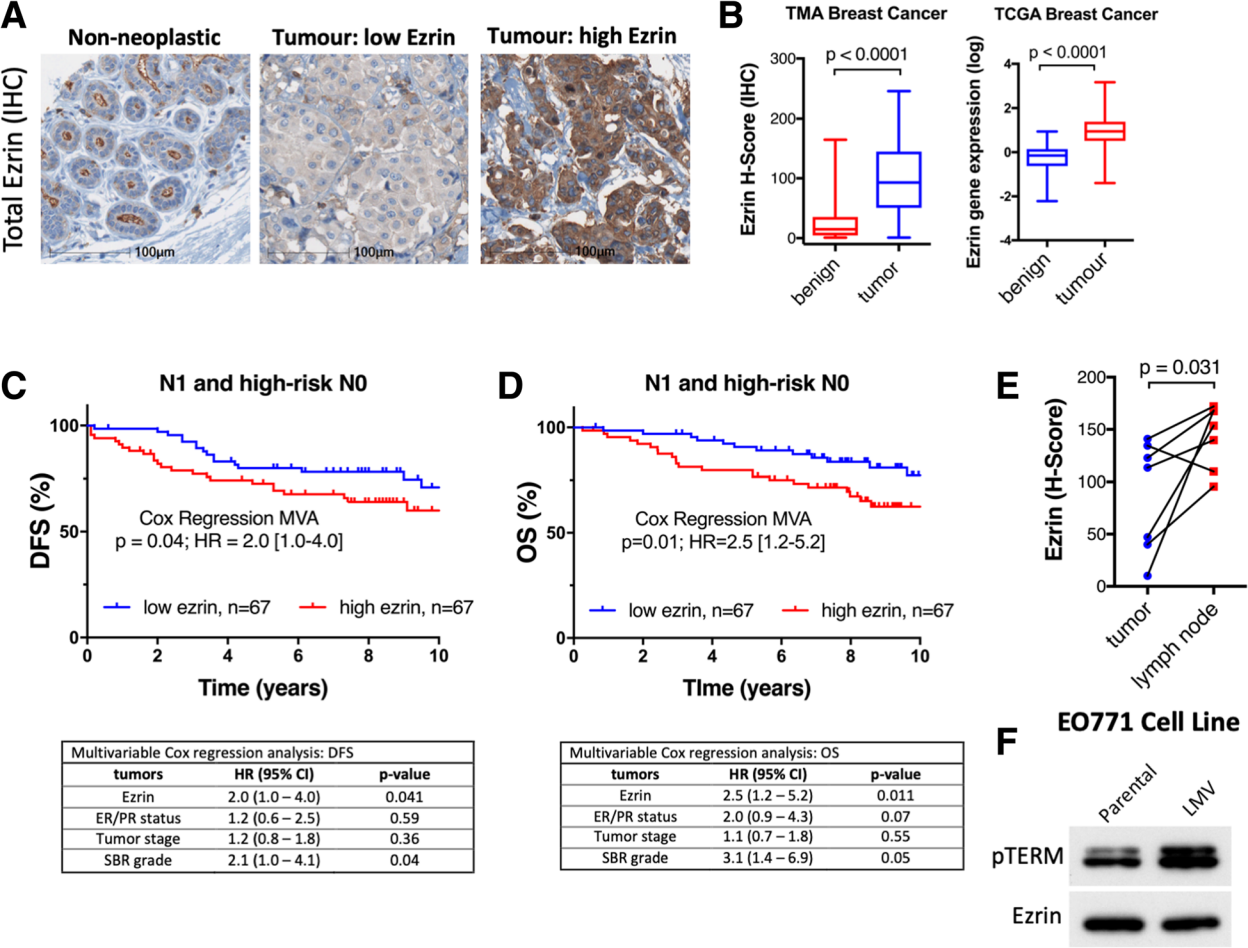
Tumor ezrin levels predict increased risk of relapse in high-risk BC patients. **a** Immunohistochemistry (IHC) of ezrin expression in non-neoplastic breast tissue and tumours with low and high ezrin are shown. **b** Box and whisker plots of ezrin histo (H)-score (protein levels, TMA) and relative *EZR* mRNA expression (TCGA) in benign and tumor tissues (*p* values from Wilcoxon matched-paired rank test). **c**, **d** KM plots showing DFS in node positive (N1, panel C) or node positive plus high-risk node negative (N0, panel D) BC patients stratified by median ezrin score. The corresponding 14 multivariate Cox regression analyses (MVA), adjusted for tumour stage, **S**carff-**B**loom-**R**ichardson (SBR) grade, and ER/PR status) are shown below each plot. **e** Ezrin expression (HALO H-score) in paired primary tumour and lymph node metastases is shown (n=7, Wilcoxon matched-pairs signed rank test). **f** Immunoblot showing elevation of phospho-ezrin (pTERM, activated ezrin) in metastatic variant cell line (LMV) derived from the murine parental cell line EO771 during serial orthotopic injections of lung metastases in C57BL/6 mice. HR, hazard ratio; CI, confidence interval

To assess the association between ezrin and risk of metastasis in BC, we quantified ezrin protein expression in primary tumors (*n* = 347, Additional file 1: Figure S1A) and a subset of matched benign ductal tissues (*n* = 90) in TMA cores, using immunohistochemistry (IHC) and HALO automated quantitative image analysis (Fig. 1a). Ezrin scores show strong core-to-core reproducibility in our TMA (Additional file 1: Figure S1B). Total ezrin protein levels showed an average of 4-fold increase in breast tumors compared to matched benign ductal tissues (Fig. 1b). Ezrin gene (*EZR*) expression was also significantly elevated in breast tumor compared with benign tissues (Fig. 1b). As ezrin plays a critical role in cancer cell invasion, we next explored its prognostic potential in patients with higher risk of metastatic disease. In multivariate survival analyses, elevated expression of ezrin in primary tumor (median used as cutoff) was associated with increased risk of relapse (DFS; HR = 2.0 (95% CI 1.0–4.0), *p* = 0.04) and mortality (OS; HR = 2.5 (95% CI 1.2–5.2), *p* = 0.01) in node-positive and high-risk node-negative patients. Survival analyses of unselected BC patients also suggested an association between elevated tumor ezrin levels and increased risk of relapse (Additional file 1: Figure S1C). Tumor ezrin levels were not prognostic in node-negative BC (Additional file 1: Figure S1C). Analysis of a small subset of matched primary tumor and LN-positive lesions from the same patient revealed significantly higher ezrin expression in LN metastases (Fig. 1e). Interestingly, higher levels of phospho-ezrin (pTERM, activated ezrin) were also observed in the metastatic variant cell line compared with the parental EO771 cells (Fig. 1f). These findings further support a role for ezrin in cancer cell invasion and as a potential marker of cancer progression and relapse in high-risk BC.

### Development of an intravital imaging model to study the effects of ezrin-targeted therapy on cancer cell migration in LN metastases

**Fig. 2:**
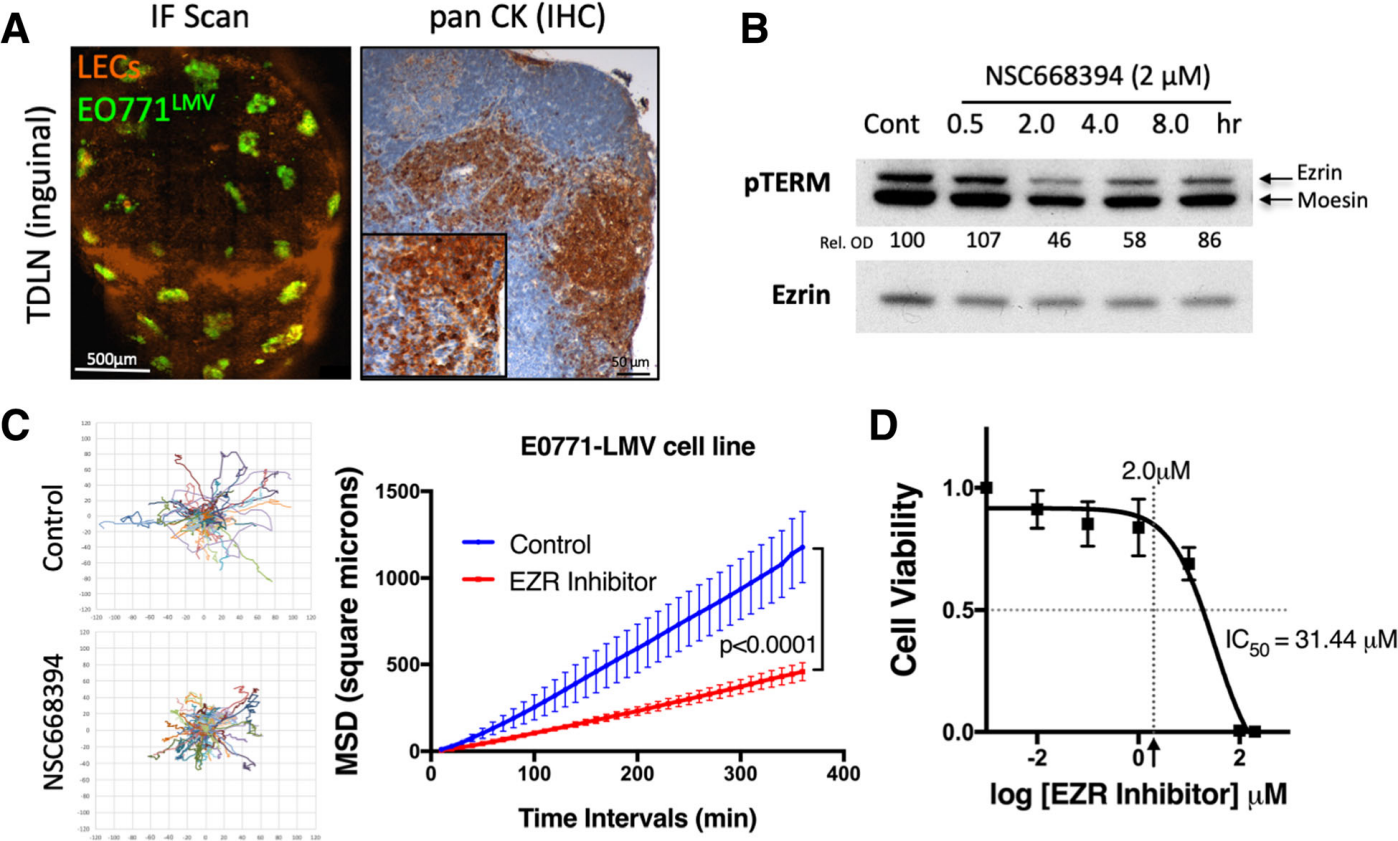
GFP-EO771^LMV^ tumors develop spontaneous LN metastases in subcutaneous and orthotopic models. **a** Whole tissue confocal scan of inguinal TDLN (left, scale bar 500 μm) and cytokeratin stain (right) confirming presence of GFP-EO771^LMV^ metastases. **b** Immunoblot analysis of GFP-EO771^LMV^ cells treated with NSC668394 ezrin inhibitor (2 μM) in vitro show reduction in ezrin pT567 (upper pTERM band). The percent ratio of phospho-ezrin to total protein normalized to control (relative optical density (Rel. OD)) shown under each band (mean of *n* = 2 assays). **c** Migration of GFP-EO771^LMV^ cells in response to ezrin inhibitor in vitro analyzed by time-lapse microscopy for up to 18 h (see Additional file 3: video 1). Cell trajectories (left, minimum of 30 cells/group, pooled from three independent assays) were used to plot mean square displacement curves (right panel) using DiPer software (*p* < 0.0001, Wilcoxon matched-paired signed rank test). **d** Cell viability analysis shows the half maximal inhibitory concentration IC_50_ in NSC668394 treatment of GFP-EO771^LMV^ cells. Th arrow points to 2.0 μM value on the *x* axis (mean of three independent assays).

The association between elevated ezrin expression and increased risk of metastases in node-positive BC prompted us to investigate the effect of pharmacological inhibition of ezrin to restrain cancer cell migration in vivo. We generated a highly metastatic cancer cell line (GFP-EO771^LMV^) from lung metastatic nodules following engraftment of the GFP-EO771 murine mammary carcinoma cells into wild-type C57BL/6 mice. Next, we developed a qIVM model to directly visualize metastatic cancer cell migration within the tumor-draining inguinal LN in syngeneic tumors engrafted into lymphatic reporter prox1-mOrange2 mice [22] (Additional file 2: Figure S2). As orthotopic mammary fat pad tumors commonly engulf the entire inguinal node in mice, we used a subcutaneous model for optimal intravital imaging of LN metastases. We observed LN metastasis in all tumor-bearing mice in our model and metastatic lesions were primarily found in the cortex region near the subcapsular sinus (SCS) of the inguinal LN (Fig. 2a). To target ezrin activity in vivo, we used a novel small molecule inhibitor (NSC668394) described previously by Bulut et al. in an osteosarcoma model [12]. GFP-EO771^LMV^ cells express ezrin and display marked reductions in phospho-ezrin pT567 level (Fig. 2b) and in-vitro migration capacity (Fig. 2c, Additional file 3: video 1 and Additional file 4: video 2) when treated with NSC668394 at concentrations (2.0 μM) well below the IC_50_ value (Fig. 2d). Migration efficiency of ezrin-deficient MDA-MB-231 cells treated with NSC668394 was not affected in comparison to their wild-type counterpart, further supporting the specificity of the inhibitor (Additional file 5: Figure S3A, B). Ezrin inhibitor had no significant effect on the rate of mitosis in MDA-MB-231 cells (Additional file 5: Figure S3C).

### Systemic treatment with an ezrin inhibitor reduces migration of cancer cells in vivo

**Fig. 3:**
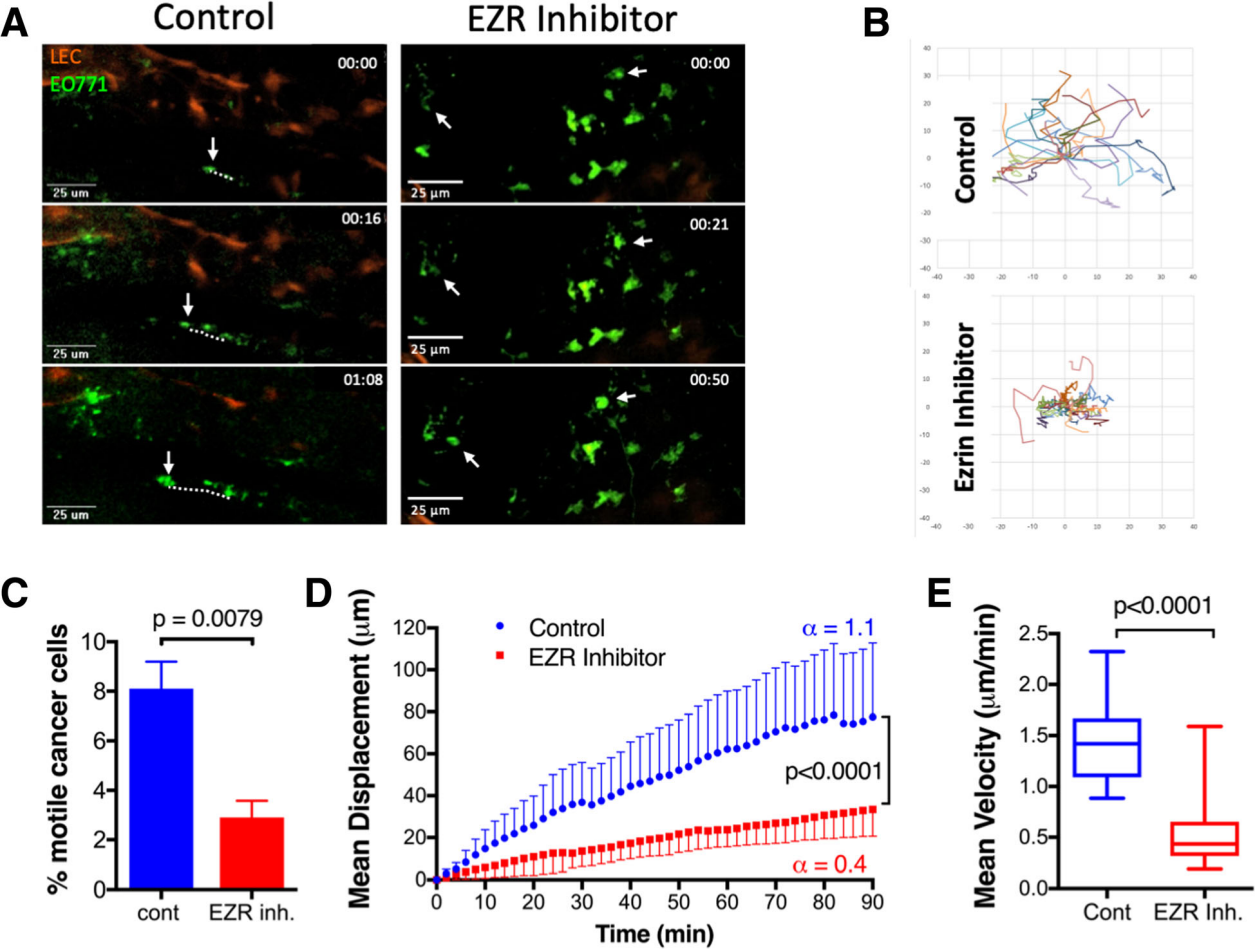
Intravital imaging of TDLN metastases reveals reduced migration of cancer cells in response to ezrin inhibition. **a** Maximum intensity projection (z-stacks of 4–6 μm) of still images from the time-lapse videos (see Additional file 6: video 3 and Additional file 7: video 4) showing migration of cancer cells (dotted line) in the inguinal LN. **b** Cell trajectory data generated for a minimum of 20 cells per group (3 fields per LN, 3 mice, pool of n=2 IVM studies; motile cells tracked by two observers blinded to the study). **c** The proportion of motile GFP-EO771LMV cells in TDLNs of control and NSC668394 treated mice (Mann Whitney test). **d** Mean displacement curves were generated from pooled cell trajectory data in panel B. The slopes of displacement curves (α-value) are shown beside each curve and compared using the Extra-Sum-of-Squares F test. **e** Overall mean velocities of motile cancer cells in control and treated TDLNs are shown (Mann Whitney test)

Using our qIVM lymphatic reporter model, we next examined the effect of systemic ezrin inhibition on cell migration dynamics within LN metastases. On day 20 post tumor injection, mice received two sequential doses of NSC668394 (0.5 mg/kg, i.p.) or vehicle (0.01% DMSO/PBS) alone at 24 h and 6 h prior to imaging (Additional file 2: Figure S2). We captured simultaneous time-lapse images from multiple sites in each ipsilateral inguinal LN for up to 2.5 h (Fig. 3a, Additional file 6: video 3 and Additional file 7: video 4). The trajectories (normalized to the origin) of all motile cells were tracked as shown in Fig. 3b. On average, about 8% of cancer cells per field of view were actively migrating within TDLNs in the untreated group, and this percentage decreased significantly in NSC668394-treated mice (Fig. 3c). The slope of the mean displacement curve was significantly reduced in motile cancer cells in mice treated with NSC668394 compared to cells in untreated mice, suggesting a reduction in their migration efficiency (Fig. 3d). Motile cells in untreated TDLNs displayed short bursts of solitary amoeboid-like movements with speeds reaching 2.5 μm/min and a mean velocity of 1.44 ± 0.4 μm/min. Ezrin inhibition did not affect the single-cell mode of motility, but cells displayed a more constrained movement with reduced top speed (1.5 μm/min) and a mean velocity of 0.56 ± 0.3 μm/min (Fig. 3d, e). Although EO771 cells are capable of a mesenchymal-like (spindle-shape) mode of migration (see Additional file 3: video 1 and Additional file 4: video 2), motile cancer cells in TDLN displayed only a single-cell amoeboid-like (rounded) mode of migration, with no evidence of collective or mesenchymal-like motility (data not shown).

### Ezrin inhibition reduces metastatic burden in the axillary LN and lungs

**Fig. 4:**
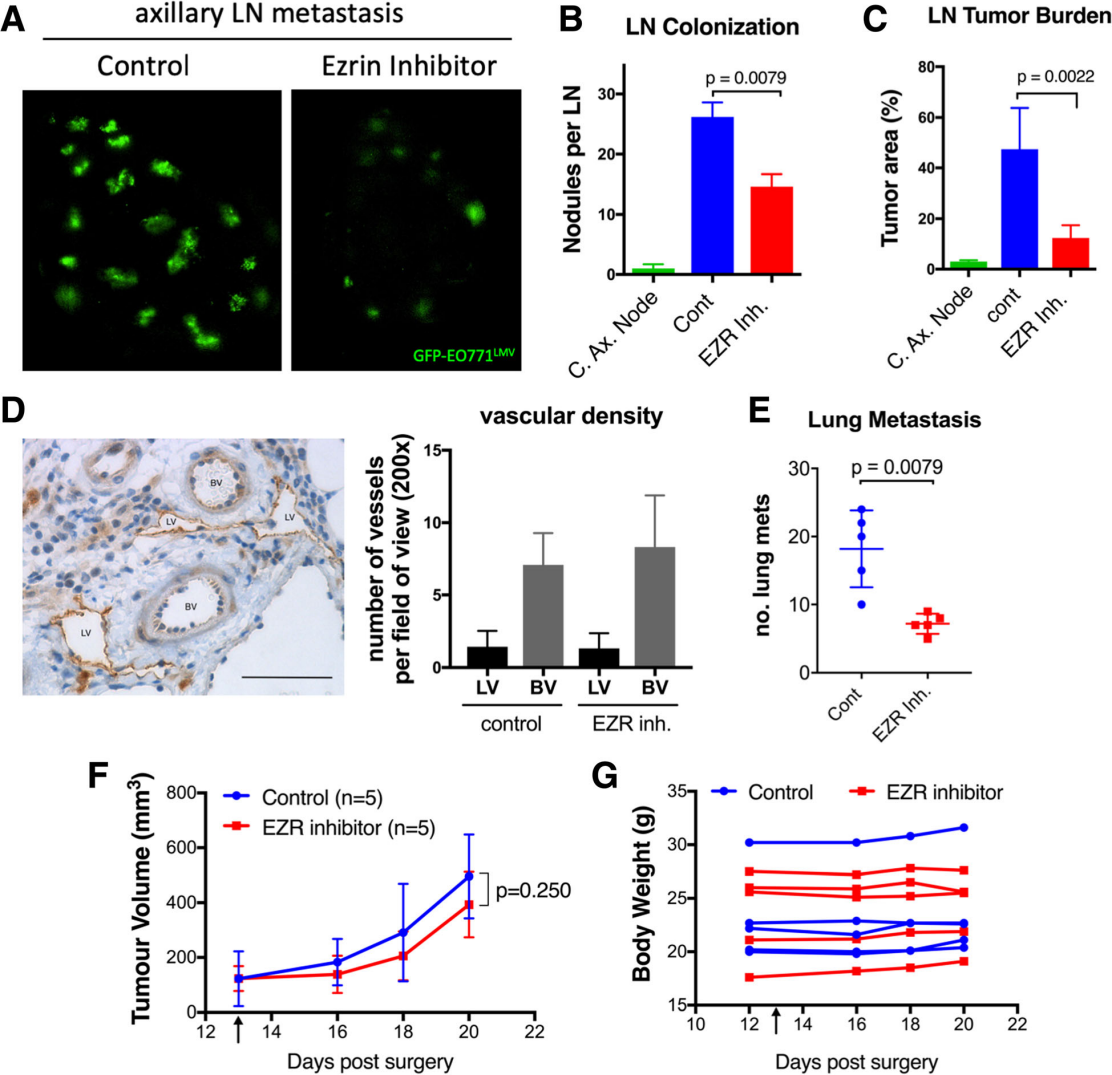
Ezrin inhibition attenuates metastatic burden in axillary LN and lungs. **a** On day 20 post injection of GFP-EO771^LMV^ cells into left fourth mammary fat pad of lymphatic reporter mice (five mice per group, *n* = 2), ipsilateral axillary LNs were harvested and scanned by confocal fluorescent microscope. **b** The total number of LN tumour nodules and **c** tumor burden (tumor area/total LN area) in contralateral axillary node (C. Ax. Node) and control and ezrin inhibitor treated ipsilateral axillary nodes are shown (p values calculated by Mann-Whitney test) **d** An example of LV (high lyve-1) and BV (low lyve-1) in intra-tumour region is shown. Scale bar in IHC image represent 50μm. The graph shows lymphatic (LV) and blood (BV) vascular 15 density assessed by visual count of at least 5 random fields of view (20x objective) in tumours stained with lyve-1 (LV marker) and CD31 (BV marker). **e** To assess lung metastasis, primary tumours were surgically removed on day 20 and mice allowed to recover for an additional 7 days. Lungs were then harvested and assessed for number of tumour nodules by fluorescence imaging (n=6 per group; p value calculated by Mann-Whitney test). **f**, **g** Primary tumour volume and total body weight of mice were measured during the study. Tumour growth curves were compared by Wilcoxon matched-paired signed rank test. Black arrow indicates the start of NSC668394 treatment

We next examined whether treatment with the ezrin inhibitor would reduce tumor colonization and metastatic burden in the distal node (axillary) and lungs in an orthotopic mammary fat pad tumor engraftment model. Mice were injected daily with NSC668394 (0.5 mg/kg, i.p.) or DMSO/PBS (control) for 7 days after GFP-EO771^LMV^ tumors reached a palpable size (~ day 13). When tumors in the control group reached ~ 500 mm^3^, we harvested axillary nodes and lungs and scanned them by confocal fluorescent microscope to quantify the metastatic load (Fig. 4a). Ezrin inhibition reduced the number of tumor colonies per node and metastatic burden by 50% (Fig. 4b) and 70% (Fig. 4c), respectively. Minimal metastases were observed in contralateral axillary nodes (Fig. 4b, c). Mice in the ezrin inhibitor-treated group also exhibited significantly lower numbers of total lung metastases compared to the control group (Fig. 4d). Assessment of lymphatic and blood vascular density of primary tumors did not reveal a significant difference between control and ezrin inhibitor-treated groups (Fig. 4e). The primary tumors were considered size-matched as we did not observe a significant difference in primary tumor size between the two treatment groups (Fig. 4f). Mice treated with NSC668394 showed no significant difference in body weight (Fig. 4g), food intake, or physical activity (data not shown). Knockdown of ezrin in an MDA-MB-231 orthotopic tumor model also resulted in significant reductions in axillary LN and lung metastases (Additional file 8: Figure S4).

## Discussion

In the present study, we have identified ezrin as an independent prognostic marker for relapse in node-positive and high-risk node-negative BC patients. Together, with elevated ezrin expression in LN metastases compared to matched primary tumors, these data support our contention that ezrin overexpression confers a metastatic advantage to BC cells during tumor progression. To study the effect of ezrin inhibition on cancer cells in vivo, we have developed a novel intravital model using a lymphatic reporter mouse to directly track cancer cell migration within lymph node metastatic nodules. A growing body of evidence implicates ezrin as a key promoter of the metastatic process in solid tumors (reviewed in [3]). Ezrin plays a critical role in maintaining epithelial integrity and in focal adhesion and invadopodia turnover, both key processes in metastatic progression of cancer cells [2, 20, 24, 25]. Data from our laboratory and other groups have shown that ezrin overexpression in breast cancer cells increases cell scattering and invasion [26, 27], whereas knockdown or mutational inactivation of ezrin reduces cancer cell migration and diminishes the metastatic potential of cancer cells [20, 28–30]. The data presented here, to our knowledge, are the first to show that pharmacological inhibition of ezrin can effectively impede migratory capacity of cancer cells in vivo and reduce metastatic burden in LNs and lungs in BC models.

Metastasis is a complex process involving tumor cell motility, intravasation, circulation in the blood or lymph system, extravasation, and growth in distant sites [31]. The increase in metastatic cancer cell motility and invasiveness is a prerequisite for cancer progression and metastasis. Therefore, one can predict that a decrease in cancer cell motility in ezrin inhibitor-treated mice would contribute to the reductions in LN and lung metastatic burden observed in this study. However, it is important to acknowledge the existence of non-ezrin-dependent mechanisms of cell migration that may play an important role in metastatic progression. The elongated mode of cell motility (or mesenchymal-like motility) has been shown to be associated with Rac-dependent F-actin-rich protrusions independent of ezrin function [32]. We cannot exclude the contribution of alternate modes of cancer cell motility in our model. In fact, we do not observe complete inhibition of cell migration in vitro or full block of distant metastases in vivo following ezrin knockdown or inhibition, suggesting that non-ezrin-dependent pathways also play a role in our model. Furthermore, clinical evidence suggests that increased lymphatic vascular density in primary tumors is associated with increased lymphatic metastasis and poor outcome in BC [5]. We have previously shown that ezrin knockdown in breast cancer cells leads to reduced lymphangiogenic activity in a Matrigel plug xenograft model in mice [8]. However, in the present study we did not observe a reduction in peritumoral vascular density in mice treated with ezrin inhibitor. It is plausible that ezrin knockdown targets functions that are independent of the p-T567 (pTERM) activation site (Fig. 2b) effected by ezrin inhibitor. Recently, Celik et al. have described a novel role for ezrin in the regulation of transcription factor DDX3 that is independent of ezrin’s activation and membrane-localized open conformation initiated by phosphorylation of T567 site. This could suggest that decreasing migration capacity of cancer cells in the presence of ezrin inhibitor could play a more prominent role in reducing distant metastasis than contribution from alterations in peritumoral vascular density in our model. Further studies are required to examine the exact mechanism of action for NSC668394 ezrin inhibitor in metastatic cascade.

The relevance of LN metastasis in the progression of metastatic disease has been a subject of considerable debate, partly due to limited models to study the dynamics of cancer cell invasion and response to anticancer therapy within metastatic sites [33, 34]. The intravital model presented in this study provides direct evidence that a subset of metastatic cells maintain their invasive capacity within LN metastases. Our observations are further supported by two recent studies on the fate of metastatic cells within LNs. Pereira et al. traced the fate of cancer cells expressing a photoconvertible protein and reported that a fraction of metastatic cells were able to invade the LN blood vessels, enter the blood circulation, and colonize the lungs in tumor-bearing mice [35]. Brown et al. also demonstrated that cancer cells microinfused into mouse afferent lymphatic vessels were able to disseminate via LN blood vessels and metastasize to the lungs without involvement of the thoracic duct [36]. Together, these observations in mouse models provide evidence that LN metastases can be a source of cancer cells for distant metastases and should therefore be part of the treatment protocol to prevent cancer progression and eliminate all disease from the patient. In fact, findings from a number of clinical trials support this theory by showing reduced rates of locoregional and systemic recurrence following addition of regional node radiation therapy to the standard of care in node-positive BC patients [6, 7].

Despite the emergence of new prognostic biomarkers and genomic profiling, the nodal status remains a key factor in BC prognosis and has critical therapeutic implications. Moreover, the incidence of lymph node-negative invasive BC has been on the rise due to advances in early detection technologies [37]. This fact, combined with less aggressive surgical biopsy and treatment of localized disease [38] plus the recent evidence that systemic spread is an early event in BC [39], highlights a need for novel prognostic factors for relapse and improved therapeutics to prevent spread of invasive cancer cells and occult micrometastases [40] in high-risk node-negative patients. The clinical evidence in our study points to ezrin as a potential independent prognostic marker for relapse in high-risk node-negative and node-positive BC. It is tempting to speculate that high-risk BC patients with elevated tumor ezrin levels could benefit from adjuvant ezrin-targeted therapy. Blocking cancer cell dissemination by ezrin-targeted therapy could also be beneficial in certain neoadjuvant settings, where therapies such as surgery, chemotherapy, or radiation have been shown to induce cancer cell motility leading to higher numbers of circulating tumor cells [41]. Moreover, Karagiannis and colleagues have recently shown that neoadjuvant chemotherapy increases the risk of metastatic dissemination, despite decreasing the primary tumor size, in a mechanism driven by upregulation of actin-regulatory protein Mammalian-enabled (MENA) in cancer cells. Authors were able to reverse the chemotherapy-induced metastatic activity by knockdown of MENA [42]. Interestingly, ezrin acts as a protein kinase A anchoring protein (AKAP) in regulating the phosphorylation of MENA, and silencing of ezrin has been shown to inhibit MENA’s function [43]. Finally, as a low tumor ezrin level is associated with improved DFS in node-positive and high-risk node-negative BC, a less aggressive treatment regimen may be warranted in these patients to improve quality of life.

## Conclusions

The effect of ezrin targeted therapy in reducing cancer cell motility, together with the clinical association of ezrin with increased risk of relapse, points to ezrin as an important regulator of the metastatic process in BC. We therefore propose that tumor ezrin levels in BC, and most likely in other cancers of epithelial origin, would act as an independent biomarker in predicting relapse and recommend further development of therapeutic approaches to target ezrin in patients with high tumor ezrin levels.

## Additional files

Additional file 1: **Figure S1.** Ezrin expression in the Southeastern Ontario Breast Cancer (SEOBC) cohort (TIFF 2523 kb)

Additional file 2: **Figure S2.** Experimental design and treatment regimen (TIFF 2020 kb)

Additional file 3: **Video 1.** Migration characteristics of GFP-EO771^LMV^ cells in vitro (MOV 9130 kb)

Additional file 4: **Video 2.** Ezrin inhibitor reduces migration of cancer cells in vitro (MOV 9838 kb)

Additional file 5: **Figure S3.** Ezrin inhibitor has no effect on migration of ezrin-deficient cells (TIFF 1731 kb)

Additional file 6: **Video 3.** Intravital imaging reveals a subset of metastatic cancer cells that retain their migratory phenotype within the TDLN (MOV 2706 kb)

Additional file 7: **Video 4.** Ezrin inhibition blocks the migration of metastatic cancer cells within the TDLN (MOV 12837 kb)

Additional file 8: **Figure S4.** Ezrin knockdown in primary tumor reduces axillary LN and lung metastasis in mice (TIFF 2009 kb)
