# Supplementary figures and images for: Intravital imaging reveals systemic ezrin inhibition impedes cancer cell migration and lymph node metastasis in breast cancer
Source: Breast Cancer Res. 2019 Jan 24;21:12. doi: 10.1186/s13058-018-1079-7 (PMC6345049; doi:10.1186/s13058-018-1079-7)

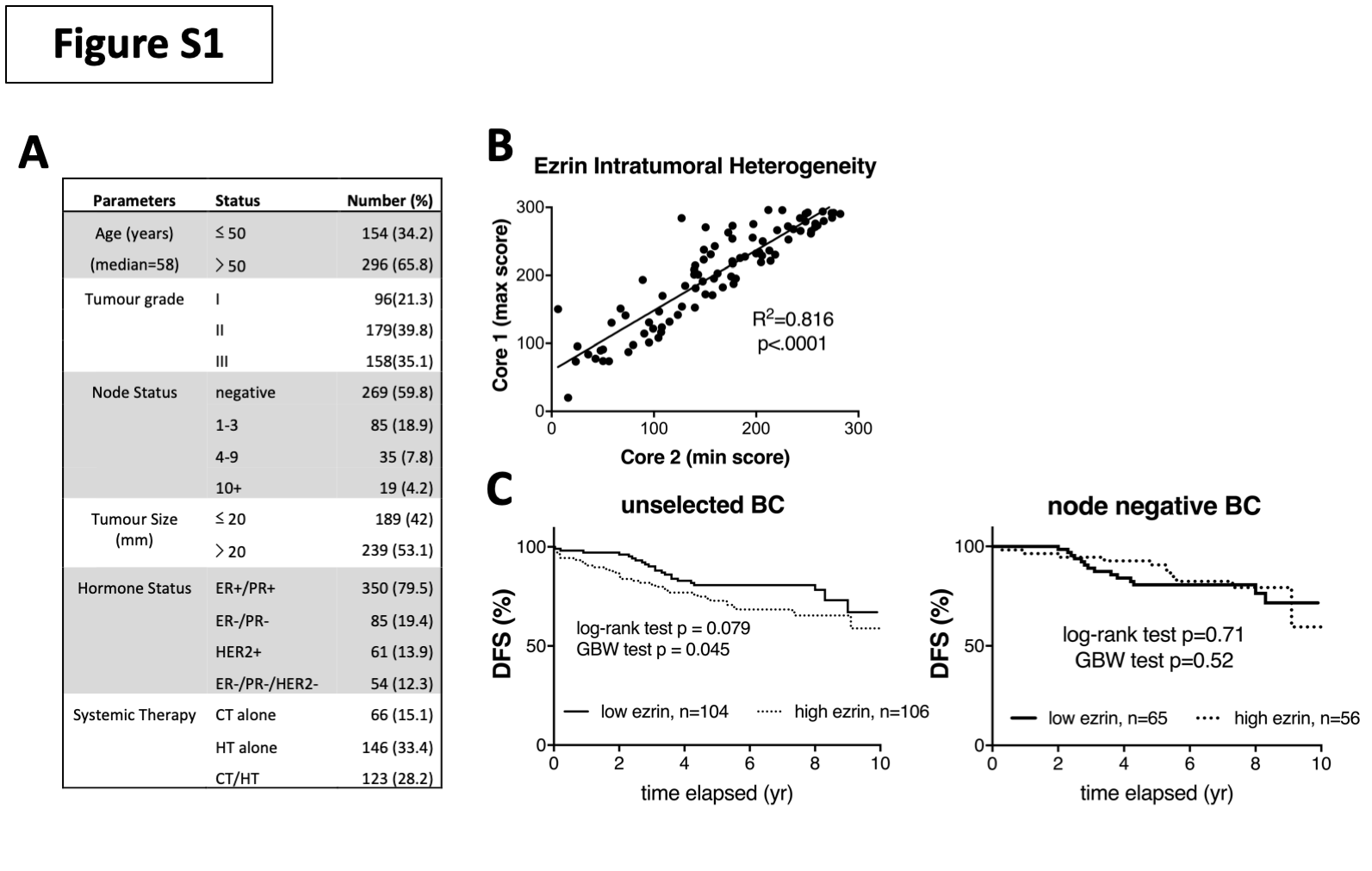

Supplement: Supplementary file 1 — Figure S1. Ezrin expression in the Southeastern Ontario Breast Cancer (SEOBC) cohort (TIFF 2523 kb) [file 13058_2018_1079_MOESM1_ESM.tiff]

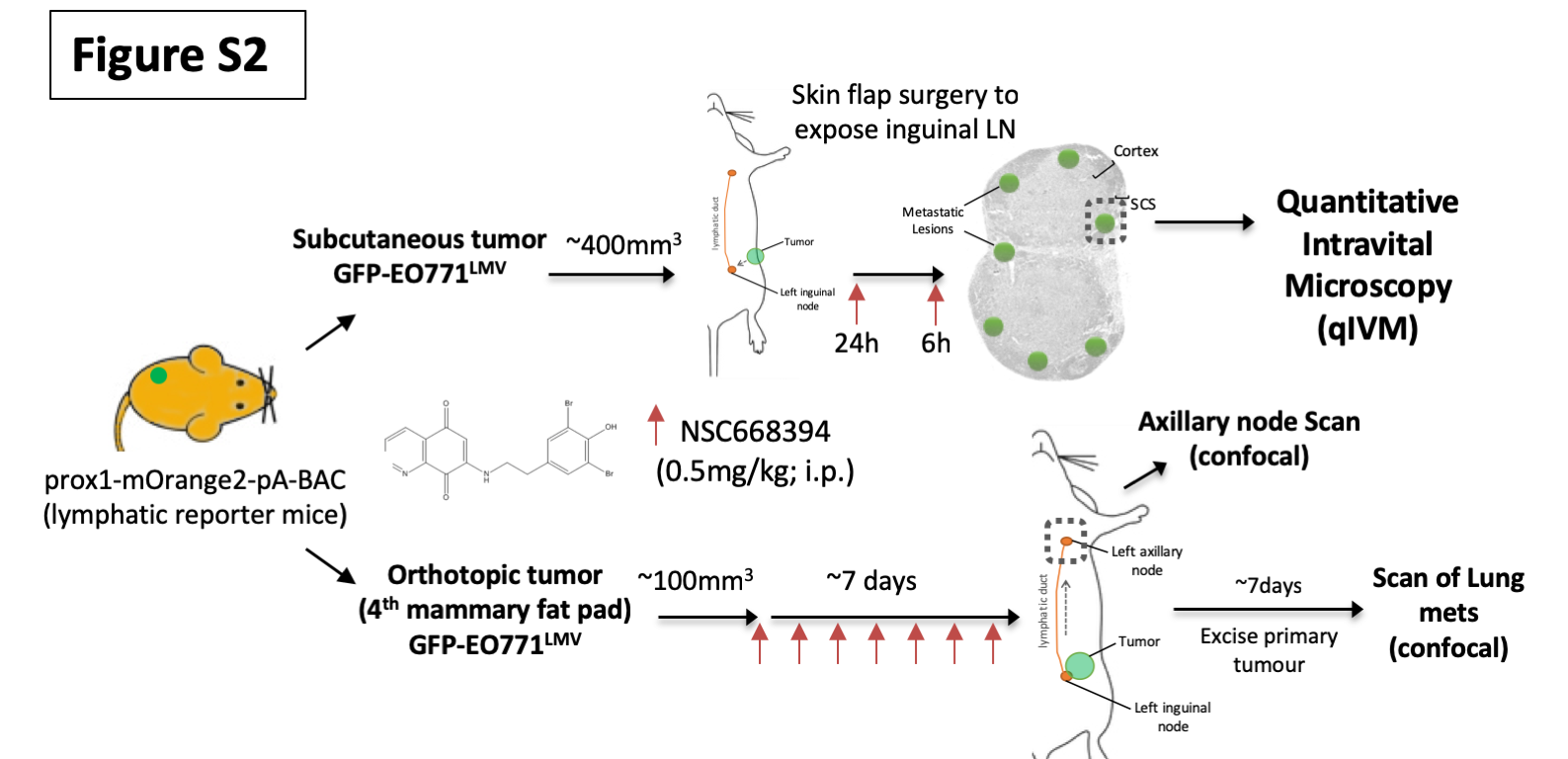

Supplement: Supplementary file 2 — Figure S2. Experimental design and treatment regimen (TIFF 2020 kb) [file 13058_2018_1079_MOESM2_ESM.tiff]

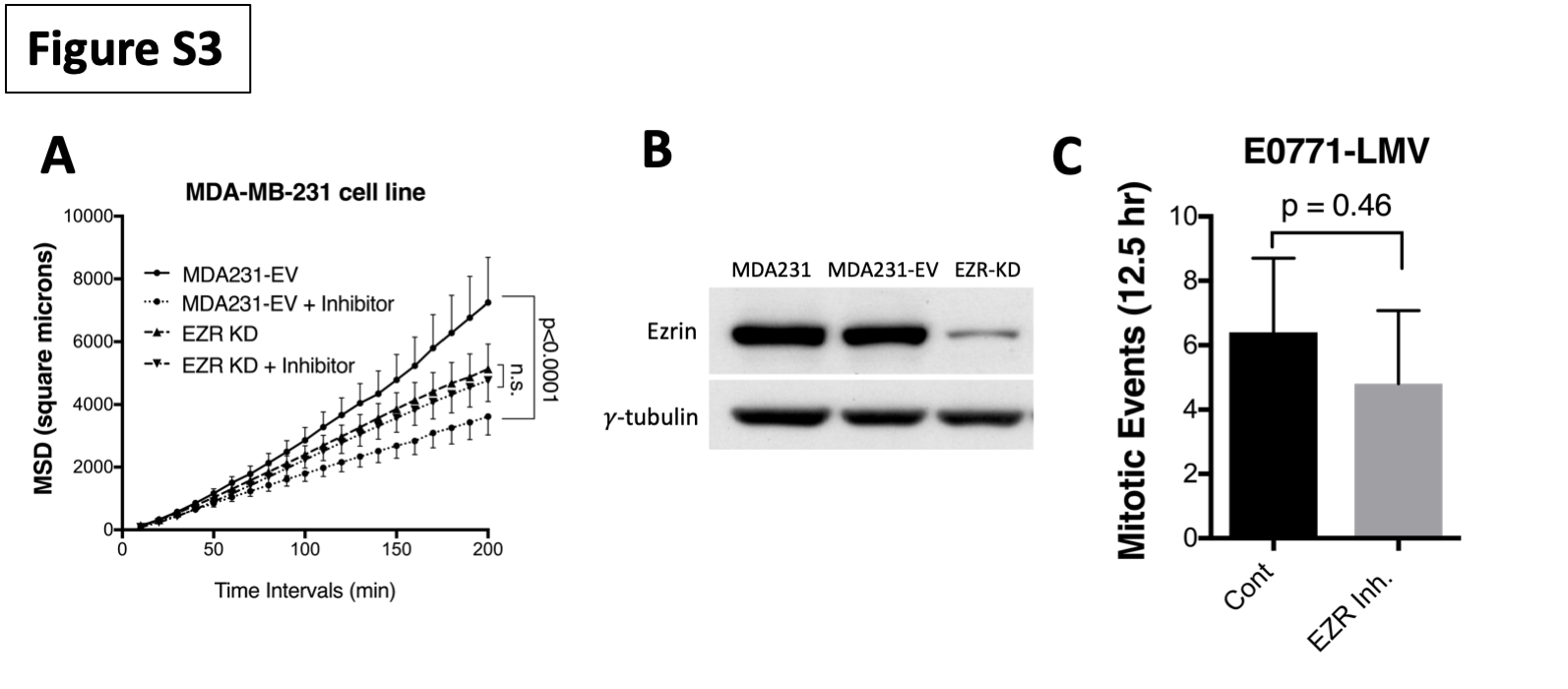

Supplement: Supplementary file 5 — Figure S3. Ezrin inhibitor has no effect on migration of ezrin-deficient cells (TIFF 1731 kb) [file 13058_2018_1079_MOESM5_ESM.tiff]

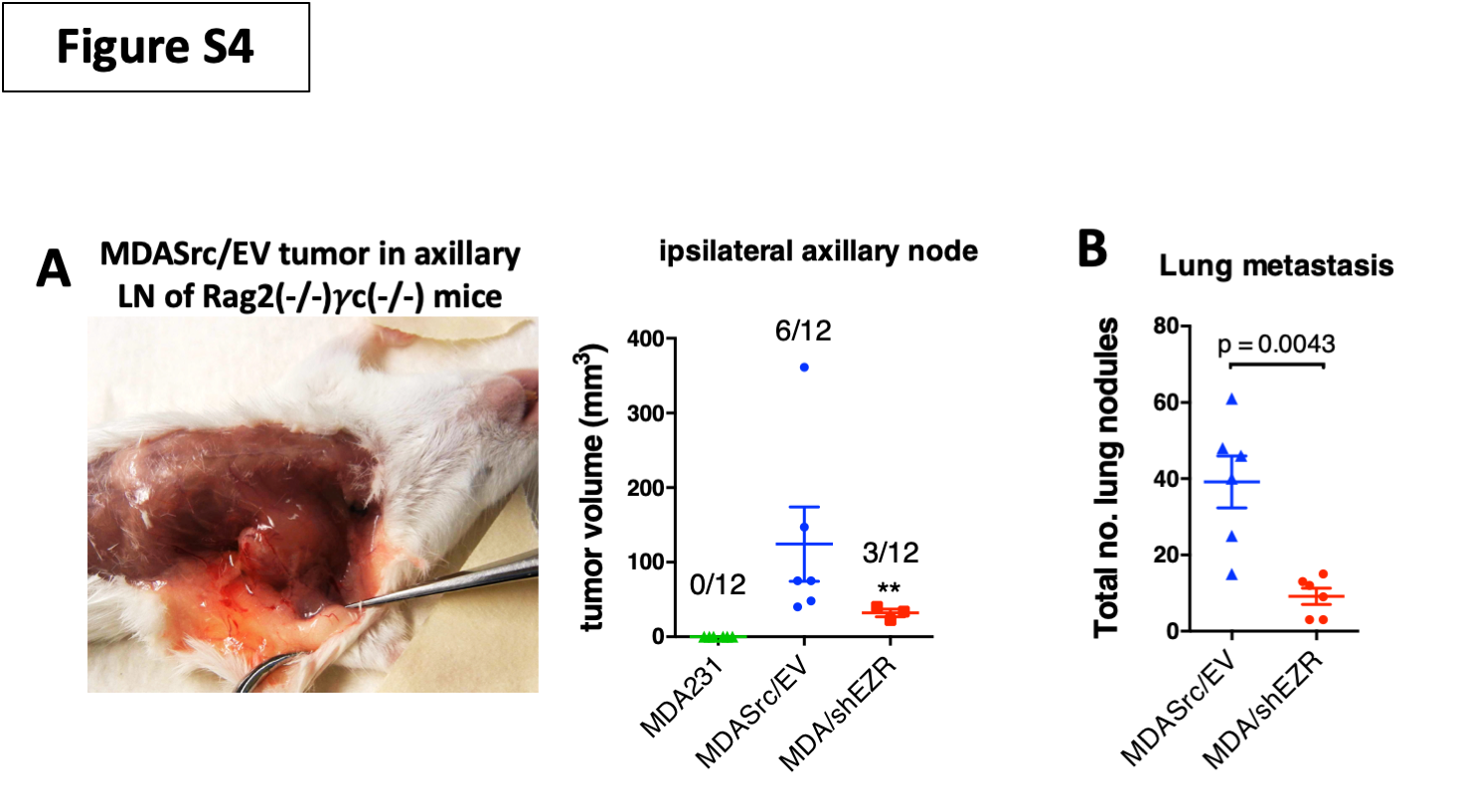

Supplement: Supplementary file 8 — Figure S4. Ezrin knockdown in primary tumor reduces axillary LN and lung metastasis in mice (TIFF 2009 kb) [file 13058_2018_1079_MOESM8_ESM.tiff]
